# Atlantool: a command line tool to retrieve DNA and RNA sequencing reads from BAM files by the read identifier

**DOI:** 10.1093/bioadv/vbaf226

**Published:** 2025-09-24

**Authors:** Emma M Rath, Huy Le, Yukhym Pyshnohraiev, Amitdev Ranjitdev, Robin Stocker, Alex Yakovlev, Congenital Heart Disease Synergy Study Group, Sally L Dunwoodie, David S Winlaw, Eleni Giannoulatou, Natasha Nassar, Edwin Kirk, Gavin Chapman, Gillian Blue, Gary Sholler, Samantha Lain, David S Winlaw, Sally L Dunwoodie, Eleni Giannoulatou

**Affiliations:** Victor Chang Cardiac Research Institute, Darlinghurst, Sydney, NSW 2010, Australia; School of Biomedical Sciences, University of New South Wales, Sydney, Kensington, NSW 2052, Australia; Atlassian, Sydney, NSW 2000, Australia; Atlassian, Sydney, NSW 2000, Australia; Atlassian, Sydney, NSW 2000, Australia; Atlassian, Sydney, NSW 2000, Australia; Atlassian, Sydney, NSW 2000, Australia; Ann & Robert H. Lurie Children's Hospital of Chicago, Chicago, IL 60611, United States; Feinberg School of Medicine, Northwestern University, Chicago, Chicago, IL 60611, United States; Victor Chang Cardiac Research Institute, Darlinghurst, Sydney, NSW 2010, Australia; School of Clinical Medicine, St Vincent's Healthcare Clinical Campus, Faculty of Medicine and Health, UNSW Sydney, Darlinghurst, Sydney, NSW 2010, Australia; Victor Chang Cardiac Research Institute, Darlinghurst, Sydney, NSW 2010, Australia; School of Clinical Medicine, St Vincent's Healthcare Clinical Campus, Faculty of Medicine and Health, UNSW Sydney, Darlinghurst, Sydney, NSW 2010, Australia

## Abstract

**Motivation:**

DNA or RNA sequencing produce a large volume of data that is usually stored in a Binary Alignment Map (BAM) file format. Processing and analysis of this large genomic data require specialized software tools. The majority of processing requirements involve accessing DNA or RNA data by chromosomal co-ordinates using SAMtools or similar software. However, challenges arise where accessing the data by the sequencing read identifier is required, and as yet there is no reliable, efficient method or tool to do this. Here we present Atlantool, a fast software that can retrieve sequencing reads from a BAM file by the read identifier. Retrieval of sequencing reads using Atlantool requires a simple command line command similar to SAMtools. After a one-time creation of a read identifier index in the same BGZF format as BAM files, retrieval of data by the read identifier appears to be instantaneous. The sequencing reads can be of any length. Atlantool fills the existing need for a reliable tool to efficiently retrieve specific records from high volume DNA or RNA sequencing data and will enable new genomic analyses to be envisaged and carried out.

**Availability and implementation:**

Precompiled Atlantool executables are freely available for download from https://github.com/VCCRI/atlantool/releases for Linux, macOS, and Windows platforms, as is a Java JAR file that permits Atlantool to run in a Java environment. The source code and user documentation are available at https://github.com/VCCRI/atlantool/.

## 1 Introduction

DNA or RNA sequencing produce large volumes of data that are usually stored in a Binary Alignment Map (BAM) file format and processed by specialized software tools, such as SAMtools (Li *et al.* 2009), BEDTools (Quinlan and Hall 2010), and Picard (Broad Institute 2019), that access DNA data by chromosomal co-ordinates. However, analysis tasks arise where accessing the data by the sequencing read identifier (qname) is required. As yet, there is no efficient method or tool to do this, and thus such analyses could not feasibly be carried out. Here we present the Atlantool software to instantaneously retrieve sequencing reads by qname read identifier from a BAM file.

## 2 The software tool

The Atlantool software is an easy-to-use command line tool that allows the user to fetch sequences from a BAM file by specifying one or more qname sequencing read identifiers, on the command line or in a file. The sequencing reads may be of any length, such as short reads (e.g. 150 base pairs) or long reads (e.g. 20 000 base pairs). Fetching of reads by the Atlantool view command appears to be instantaneous, because it uses an index. Index creation is more computationally expensive and must be run once before carrying out any fetches. The Atlantool commands are simple and are similar to SAMtools (Li *et al.* 2009):

./atlantool-linux index my_bam_file.bam

./atlantool-linux view my_bam_file.bam -n A01221:43:HVKW3DSXY:3:2506:15845:19633

./atlantool-linux view my_bam_file.bam –f my_input_file_of_qnames.txt > output.sam

## 3 Architecture

The design goal of Atlantool is to make the individual qname search fast enough (sub-second latency) for user interaction or high-volume processing. The cost of achieving this performance is the need to perform a separate indexing step, which is done once per BAM file. For a BAM file with N sequencing reads, indexing time is proportional to *N* and individual qname search time is proportional to √*N*.

The tool creates two BGZF-compressed files in a new directory named <bam_file_name>.atlantool-index during the indexing process:

- qname.v3.index.bgz
- qname.v3.data.bgz

Each of the files uses the following BGZF format:

xx  1 byte  length of qname (N)

xx…  N bytes  qname (key)

xx xx xx xx xx xx xx xx

    8 bytes  virtual offset (pointer)

The file qname.v3.data.bgz contains each qname from the original file mapped to the corresponding record position in the original BGZF-compressed BAM file. Records are sorted by qname.

The file qname.v3.index.bgz contains a subset of all qnames (approximately the square root of the full set). Each qname points to the corresponding record in the qname.v3.data.bgz file. Records are sorted by qname.

Atlantool carries out indexing by streaming the original BAM file and creating both index files. It searches for an individual qname input by performing the following steps:

1. Iterate through qname.v3.index.bgz to find the last record where qname <= input.
2. Starting from the offset from step 1, iterate through qname.v3.data.bgz to find qname = input.
3. Using the offset from step 2, look up the record in the original BAM file.

**Figure 1.**
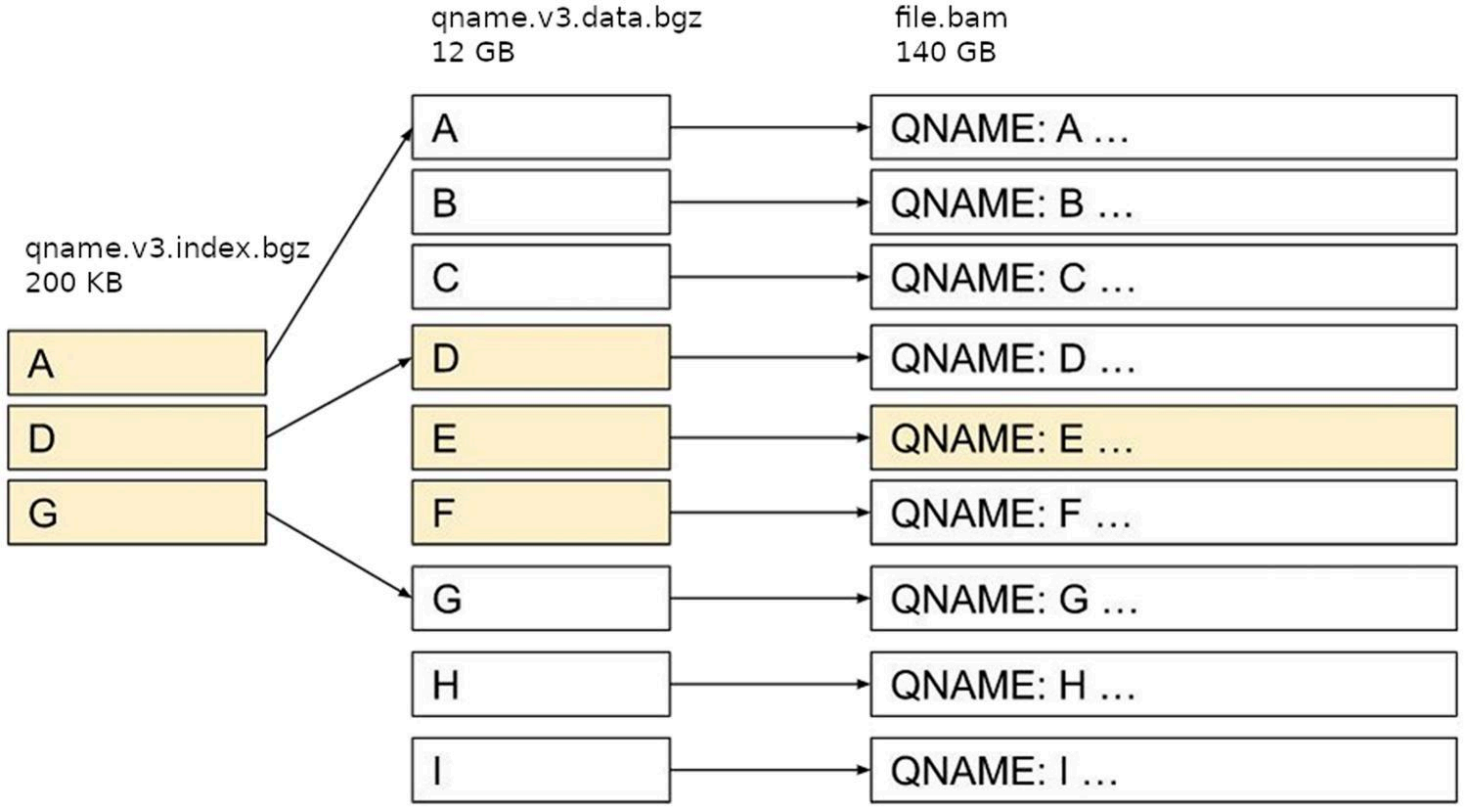
Search process for qname read identifier “E.” Scanned records required to be loaded into memory are highlighted in yellow.

The two-level indexing design allows the search algorithm to keep only the small index file qname.v3.index.bgz in memory, while minimizing the amount of data needed to be read and processed from disk from the larger index file qname.v3.data.bgz. An example of this search process is shown in Fig. 1.

## 4 Benchmarking

To comprehensively evaluate the performance of Atlantool, we benchmarked it on a diverse set of BAM files representing a range of sequencing platforms, read lengths, and file sizes. This included 187 whole genome sequencing (WGS) BAM files from the Australian Genomics Health Alliance (AGHA) Cardiovascular Flagship cohort (Austin *et al.* 2024), containing 150 bp Illumina reads sequenced to ∼30× depth, with file sizes ranging from 40 to 94 GB (mean 52 GB). We also tested 24 whole exome sequencing (WES) BAM files from the Congenital Heart Disease Synergy Study with read lengths of 150 bp and file sizes between 4.7 and 13 GB (mean 8 GB), sequenced to ∼100× depth. To assess scalability on very large datasets, we included high-coverage Illumina WGS BAM files from public datasets (Zook *et al.* 2020) such as NA24143, NA24385, and NA12878, with file sizes up to 195 GB. In addition, we benchmarked long-read data, including two PacBio BAM files (PacBio SMRT Resources 2021) (one containing HiFi reads and one standard) with file sizes of 29 and 51 GB, respectively, and average read lengths of ∼22 000 bp. We also included two Nanopore BAM files (Jain *et al.* 2018) (∼11 GB each) with read lengths of ∼13 000 bp. Altogether, these datasets span BAM sizes from 4.7 to 195 GB and demonstrate Atlantool’s applicability across short- and long-read sequencing technologies and diverse analysis contexts.

**Figure 2.**
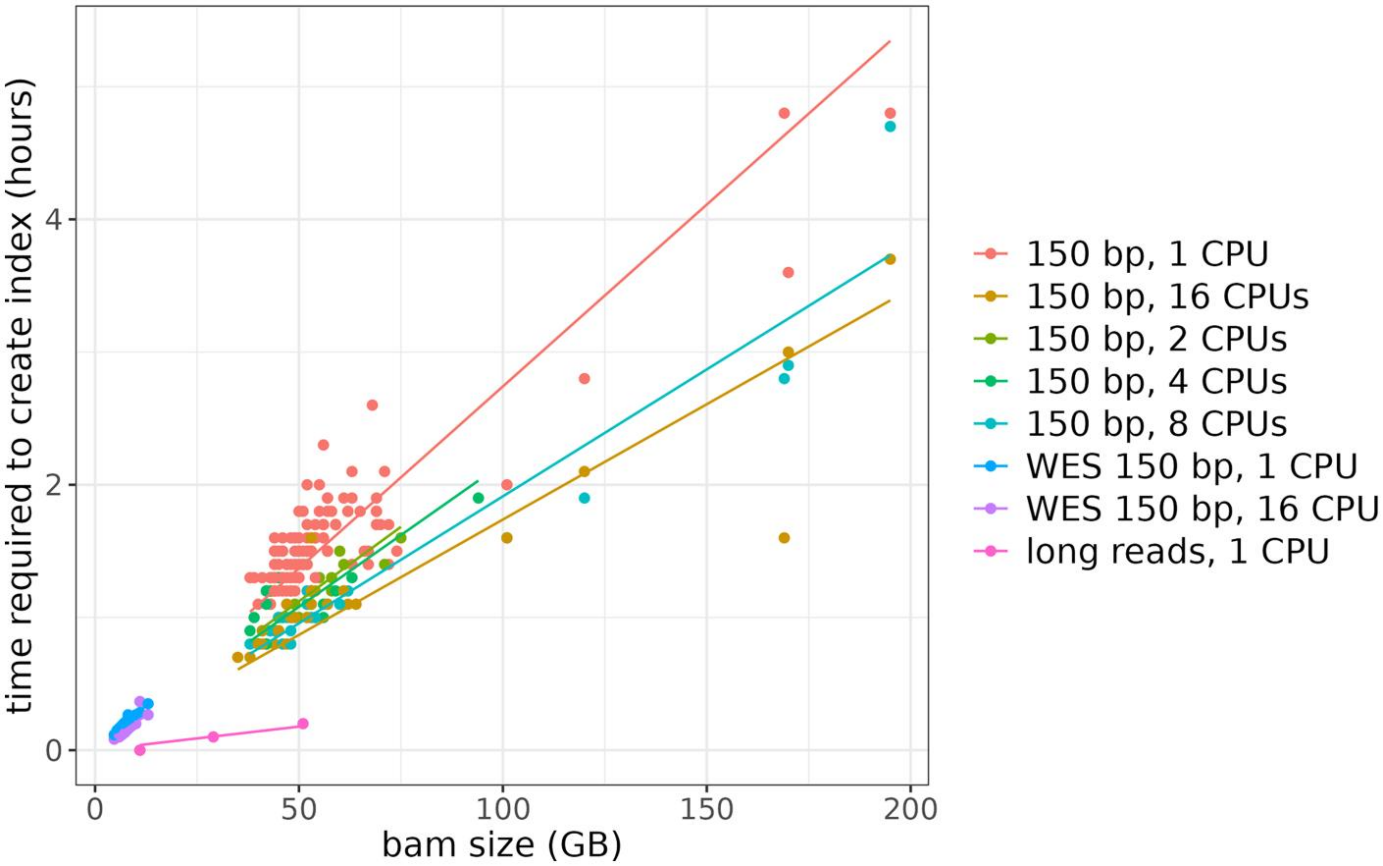
Time required to create Atlantool indexes as a function of BAM file size for short-read and long-read sequencing data, using 1–16 CPUs. BAM files represent whole genome sequencing (WGS) unless otherwise indicated as whole exome sequencing (WES). Points indicate actual measurements; lines represent fitted linear regressions.

We benchmarked Atlantool index creation across a broad range of BAM file types, sequencing technologies, and file sizes (Fig. 2 and Supplementary Fig. 1, available as supplementary data at *Bioinformatics Advances* online). For 187 Illumina whole genome sequencing (WGS) BAM files from the AGHA Cardiovascular Flagship cohort (VCGS), with average file size 52 ± 9 GB and 150 bp reads, index creation took 1.5 ± 0.3 h using 1 CPU and 8 GB RAM. This was reduced to 0.9 ± 0.1 h with 8 CPUs, with little further gain using 16 CPUs (0.8 ± 0.1 h). Index size was 6 ± 1 GB, ∼12% of the BAM file size. For 24 Illumina whole exome sequencing (WES) BAM files from the Congenital Heart Disease Synergy Study, with average size 8 ± 2 GB, index creation took 12 ± 3 min with 1 CPU and 9 ± 2 min with 16 CPUs. Index size was 1.0 ± 0.2 GB, ∼14% of BAM size. For larger Illumina WGS files (e.g. a 195 GB BAM), indexing took up to 4.8 h, with index size still ∼12% of BAM size. For long-read datasets, index creation was faster and indexes were much smaller due to fewer reads: two PacBio BAM files (∼22 000 bp reads, 29–51 GB in size) took ∼10 min to index, and index sizes were <0.05% of the BAM size. Similarly, Nanopore BAM files (∼13 000 bp reads, 11 GB each) had indexes ∼0.4% of the BAM size.

Once the index is created, Atlantool enables near-instantaneous (<1 s) retrieval of sequencing reads by qname (read identifier), regardless of the number or position of the reads requested. In contrast, equivalent retrievals using SAMtools (Li *et al.* 2009) piped to grep, or GATK's FilterSamReads (Broad Institute 2019) utility, required ∼28 min per individual query, making large-scale qname-based analyses infeasible using standard tools (Supplementary Note, available as supplementary data at *Bioinformatics Advances* online).

## 5 Use cases

Atlantool is broadly useful for applications that require efficient retrieval of sequencing reads by their read identifier (qname) from BAM files. While not typically part of standard variant-calling pipelines, qname-based retrieval is essential in many research contexts where read-level evidence is needed. These include comparing read mappings across different genome builds (e.g. to assess mapping differences when alternate contigs are present or absent), investigating viral integration events in host genomes, validating structural variants, and curating read-level discrepancies in complex datasets. Atlantool’s speed and scalability make it particularly valuable for large-scale or iterative analyses where conventional tools are prohibitively slow. We anticipate further applications in areas such as microbial or mobile element analysis, metagenomics, and data forensics, where direct access to individual reads is necessary to resolve biological or technical questions.

## 6 Conclusions

The Atlantool software enables analyses that require multiple fetching of sequencing reads by qname from DNA or RNA sequencing BAM files that were previously not feasible. Its speed and modular design make it a valuable addition to the bioinformatics toolkit for workflows requiring direct read-level evidence.

## Supplementary Material

vbaf226_Supplementary_Data
