# Supplementary material for: Atlantool: a command line tool to retrieve DNA and RNA sequencing reads from BAM files by the read identifier
Source: Bioinform Adv. 2025 Sep 24;5(1):vbaf226. doi: 10.1093/bioadv/vbaf226 (PMC12602190; doi:10.1093/bioadv/vbaf226)
Supplement: vbaf226_Supplementary_Data [file vbaf226_supplementary_data.pdf]

Supplementary Material

Atlantool: A command line tool to retrieve DNA and RNA sequencing reads from BAM files by the read identifier

Emma M Rath[1,2], Huy Le[3], Yukhym Pyshnohraiev[3], Amitdev Ranjitdev[3], Robin Stocker[3], Alex Yakovlev[3], Congenital Heart Disease Synergy Study group, David S Winlaw[4,5], Sally L Dunwoodie[1,6], Eleni Giannoulatou[1,6]

Affiliations: [1]Victor Chang Cardiac Research Institute, Sydney, Australia. [2]School of Biomedical Sciences, Faculty of Medicine and Health, UNSW Sydney, Australia. [3]Atlassian, Sydney, Australia. [4]Lurie Children's Hospital of Chicago, USA. [5]Feinberg School of Medicine, Northwestern University, USA. [6]School of Clinical Medicine, Faculty of Medicine and Health, UNSW Sydney, Australia.

Congenital Heart Disease Synergy Study group: Sally L Dunwoodie, David S Winlaw, Eleni Giannoulatou, Natasha Nassar, Edwin Kirk, Gavin Chapman, Gillian Blue, Gary Sholler, Samantha Lain.

Supplementary Note

Benchmarking of Atlantool sequencing read fetches:

In order to compare Atlantool fetch performance with available alternative methods, sequencing reads were fetched by qname from a BAM file by using SAMtools (Li et al. 2009) and grep, or by using GATK picard FilterSamReads utility (Broad 2019). The BAM file was 62 GB in size and contained 150 bp long reads of a human genome sequenced to ~30x depth. The two alternative methods' commands are below. Each of these alternative methods required 28 minutes to fetch reads by qname value, regardless of whether the reads were located on a chromosome or contig at the beginning, middle or end of the list of contigs in the BAM file.

1)
```
samtools view input_bam_file.bam | grep -f input_file_listing_qnames.txt > output_reads.sam
```

2)
```
java -jar picard.jar FilterSamReads I=input_bam_file.bam O=output_reads.sam READ_LIST_FILE=input_file_listing_qnames.txt FILTER=includeReadList
```

The same fetches by qname by Atlantool on the same platform produced results immediately.

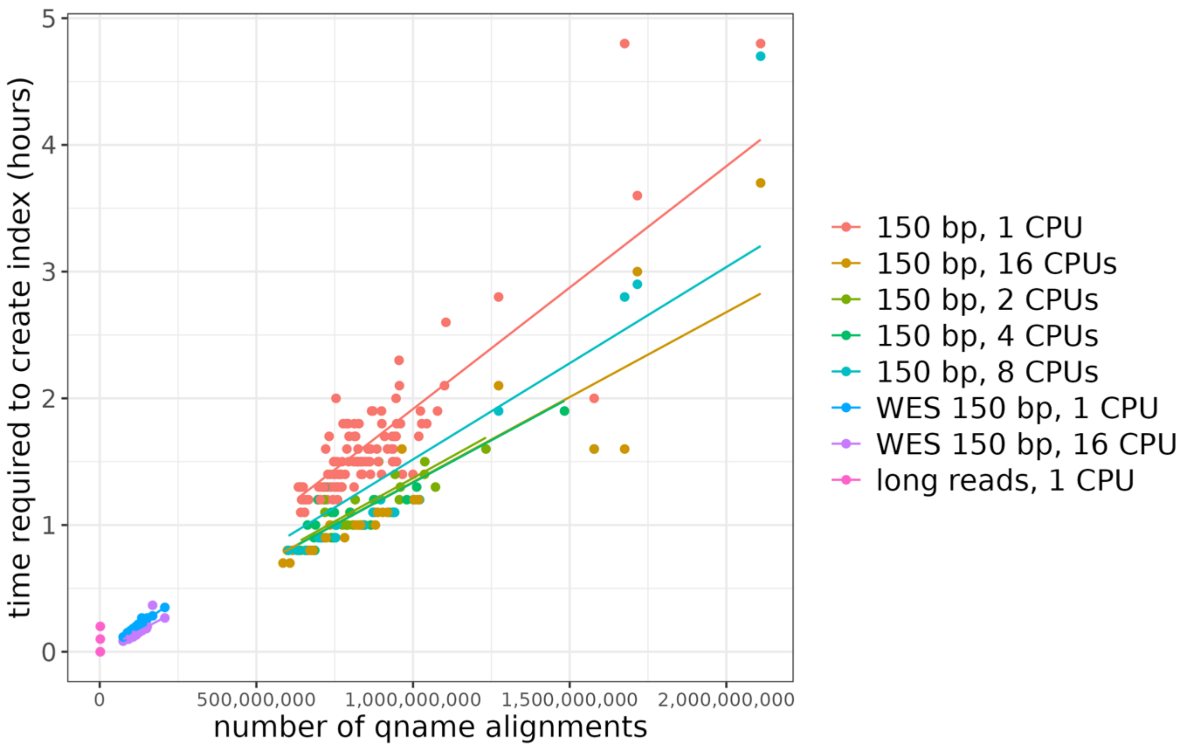

Supplementary Figure S1. Time required to create Atlantool indexes as a function of the number of reads, for BAM files having short (150 bp) or long reads, when using 1 to 16 CPUs for processing. BAM files are for whole genome sequencing (WGS) unless marked as whole exome sequencing (WES). Points are actual measurements and lines are fitted linear regressions.

References:

- Broad Institute. "Picard Toolkit." 2019. Broad Institute, GitHub Repository. http://broadinstitute.github.io/picard/
- Li H, Handsaker B, Wysoker A, Fennell T, Ruan J, Homer N, Marth G, Abecasis G, Durbin R; 1000 Genome Project Data Processing Subgroup. The Sequence Alignment/Map format and SAMtools. Bioinformatics. 2009 Aug 15;25(16):2078-9.
